# Model Updating for Nam O Bridge Using Particle Swarm Optimization Algorithm and Genetic Algorithm

**DOI:** 10.3390/s18124131

**Published:** 2018-11-26

**Authors:** H. Tran-Ngoc, S. Khatir, G. De Roeck, T. Bui-Tien, L. Nguyen-Ngoc, M. Abdel Wahab

**Affiliations:** 1 Department of Electrical Energy, Metals, Mechanical Constructions, and Systems, Faculty of Engineering and Architecture, Ghent University, 9000 Gent, Belgium; Hoa.tran@Ugent.be or ngochoa@utc.edu.vn (H.T.-N.); khatir_samir@hotmail.fr (S.K.); 2 Department of Bridge and Tunnel Engineering, Faculty of Civil Engineering, University of Transport and Communications, Hanoi, Vietnam; btthanh@utc.edu.vn (T.B.-T.); longnguyen@utc.edu.vn (L.N.-N.); 3 KU Leuven, Department of Civil Engineering, B-3001 Leuven, Belgium; guido.deroeck@kuleuven.be; 4 Institute of Research and Development, Duy Tan University, 03 Quang Trung, Da Nang, Vietnam; 5 Soete Laboratory, Faculty of Engineering and Architecture, Ghent University, Technologiepark Zwijnaarde 903, B-9052 Zwijnaarde, Belgium

**Keywords:** model updating, particle swarm optimization, genetic algorithm, large-scale bridges, stiffness of truss joints

## Abstract

Vibration-based structural health monitoring (SHM) for long-span bridges has become a dominant research topic in recent years. The Nam O Railway Bridge is a large-scale steel truss bridge located on the unique main rail track from the north to the south of Vietnam. An extensive vibration measurement campaign and model updating are extremely necessary to build a reliable model for health condition assessment and operational safety management of the bridge. The experimental measurements are carried out under ambient vibrations using piezoelectric sensors, and a finite element (FE) model is created in MATLAB to represent the physical behavior of the structure. By model updating, the discrepancies between the experimental and the numerical results are minimized. For the success of the model updating, the efficiency of the optimization algorithm is essential. Particle swarm optimization (PSO) algorithm and genetic algorithm (GA) are employed to update the unknown model parameters. The result shows that PSO not only provides a better accuracy between the numerical model and measurements, but also reduces the computational cost compared to GA. This study focuses on the stiffness conditions of typical joints of truss structures. According to the results, the assumption of semi-rigid joints (using rotational springs) can most accurately represent the dynamic characteristics of the truss bridge considered.

## 1. Introduction

Structural health monitoring and safety assessments for long-span bridges have become a dominant research topic, and have received the special attention of infrastructure authorities in recent years. Around the world, for many large-scale bridges, monitoring systems are installed to evaluate the health condition of the bridge and to guarantee operational safety management [1]. The Finite Element Method (FEM) is the standard tool for modeling structural behavior. However, due to a wide range of simplifying assumptions that rely on engineering judgment and a lot of uncertainties in structural properties (e.g., material properties, boundary conditions, etc.), the FE model is often only an approximate representation of the real structure. Therefore, the initial FE model should be corrected. If measured dynamic characteristics (such as natural frequencies, mode shapes, and modal damping ratio, etc.) are available, model updating can be applied to minimize the deviation between numerical prediction and experimental results. In recent decades, vibration-based bridge health monitoring (VBHM) has been more and more commonly used to experimentally determine the physical behavior of large-scale bridges. Wu et al. [2] made use of the measurements with spatially-distributed optical fiber sensors during static loading tests, to update an FE model of a highway bridge. The first order modal macro-strains and static long-gauge strains were included in the objective function. Feng et al. [3] updated a short-span steel railway bridge by applying a time-domain FE model updating approach based on in-situ dynamic displacements under ambient vibrations after train passage, instead of using modal parameters (natural frequency, mode shape, etc.). They concluded that in the case of short-span railway bridges, it was difficult to extract dynamic behavior (natural frequencies) from measurements after train passing, because the natural frequencies of such bridges were much higher than the frequencies of trainload’s excitation. Bayraktar et al. [4] updated a balanced cantilever bridge by a manual tuning procedure, using the peak picking method in the frequency domain. In their study, frequencies of the first 10 modes were selected as objective function. El-Borgi et al. [5] applied the enhanced frequency domain decomposition technique and the Femtools software for model updating of a reinforced concrete bridge in Tunisia. They used both frequencies and mode shapes as objective functions, and concluded that Young’s modulus of concrete decreased dramatically during its service life. Brownjohn et al. [6] updated a small highway bridge in a refurbishment and strengthening project in Singapore. They demonstrated that the calculated natural frequencies of the first eight modes increased by approximately 50% after model updating. Minshui et al. [7] applied a simple updating method to determine the physical behavior of a highway bridge. The objective function was to minimize the difference between the theoretical and experimental natural frequencies measured during ambient excitation. After model updating, the difference was reduced from 8.77% to 4.56% for the largest deviation. Parisa Asadollahi et al. [8] adapted a full-scale model of a long-span cable-stayed bridge through Bayesian FE model updating using long-term monitoring data of modal characteristics collected from a wireless sensor network during a one-year period. Zhang et al. [9], Ren et al. [10], BTemel Türker et al. [11], Teughels et al. [12], Schlune et al. [13], and many other researchers also presented examples of updating long-span bridges. 

Numerous researchers have also applied optimization algorithms for model updating, and they obtained good results for different types of structures and fields, e.g., Tiachacht et al. [14], Samir et al. [15], Khatir et al. [16], Yuan et al. [17], Hou et al. [18], and Hamdia et al. [19,20]. Regarding its application to bridges, Qin et al. [21] combined the particle swarm optimization (PSO) algorithm with a surrogate model to update higher vibration modes for a continuous railway concrete bridge in Spain. They pointed out that combining the PSO algorithm and a surrogate model reduced the computational time. They achieved a good level of accuracy between the numerical and experimental results. Jung et al. [22] applied a hybrid genetic algorithm to update a small-scale bridge. The objective function included static deflections, mode shapes, and natural frequencies. Deng et al. [23] combined the response surface method and a genetic algorithm (GA) to update a simply supported beam. The response surface was selected as an objective function, and GA was employed to find the best solution. Qin et al. [1] updated a complex railway bridge by using GA combined with the Kriging model. While the Kriging model acted as a surrogate to reduce the deviation between the structural parameters and responses, GA provided the opportunity for obtaining the global best solution. Liu et al. [24], Zordan et al. [25] and other authors also employed the mathematical power of optimization algorithms to update large-scale bridges.

This paper presents a study for updating a large-scale truss bridge. The first step is building an initial FE model, in which three possibilities of joint conditions (pin, rigid, and semi-rigid) are considered. The second step is to simulate the effect of different joint assumptions. For steel truss structures, the joint stiffness’s are the most uncertain parameters that will influence the result of dynamic analysis. Therefore, this paper applies three scenarios of truss joints, namely pin, rigid, and semi-rigid, for the Nam O Bridge, and then analyzes and selects the scenario that properly reflects the dynamic behavior. This approach is also applied by some other authors [26,27,28]. However, most of aforementioned authors only evaluated different joint assumptions for static analysis, while others analyzed the joint conditions for the dynamic analysis of scale models in the laboratory. To the best of the authors’ knowledge, analyzing and evaluating the effects of different joint assumptions has never been applied to large-scale truss bridges. In this paper, we attempt to deal with this effect for the first time. The last step is employing an optimization algorithm to update some uncertain parameters such as material properties and the joint stiffness of the bridge. PSO and GA algorithms are employed to consider the efficiency of the optimization algorithm for model updating. Both PSO and GA are evolutionary algorithms based on the stochastic optimization technique, making use of a group of a random population to find the best solution through a fitness function. In GA, the information of all the particles is shared with each other after an iteration, whereas in PSO, only the best global position of particles, i.e., the best solution, is given out. In this paper, the efficiency of both optimization algorithms is compared, based on computational cost and convergence level. 

This paper is organized as follows: after an introduction, a detailed description of the bridge is presented. The next section describes the experimental campaign and the extracted modal parameters. Afterwards the finite element model is presented, followed by a section presenting the results of the model update. Finally, conclusions are formulated.

## 2. Description of the Bridge

**Figure 1:**
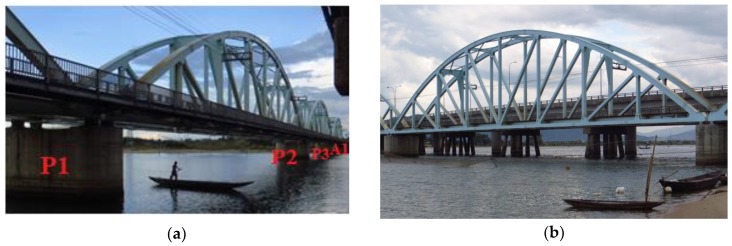
Some views of Nam O Bridge: (**a**) Upstream side; (**b**) Downstream side.

The Nam O Railway Bridge is a large-scale steel truss bridge, located in Da Nang city in the middle of Vietnam. The bridge plays a vital role in connecting train traffic from the north to the south. The Nam O Bridge is constructed in 2011, with funding from the Hanoi–Ho Chi Minh City Line Bridge Safety Improvement Project. The bridge includes four simply supported spans of equal length (75 m). The rail track is placed directly on the stringers of the bridge deck. The abutment on the Hai Van side is referred to as *A-0*, whereas the three piers are numbered as *P-1*, *P-2*, and *P-3*, starting from the *A-0* side. The last (forth) span goes from *P-3* to the abutment *A-1* on the Da Nang city side. Some views of the bridge are given in Figure 1.

**Figure 2:**
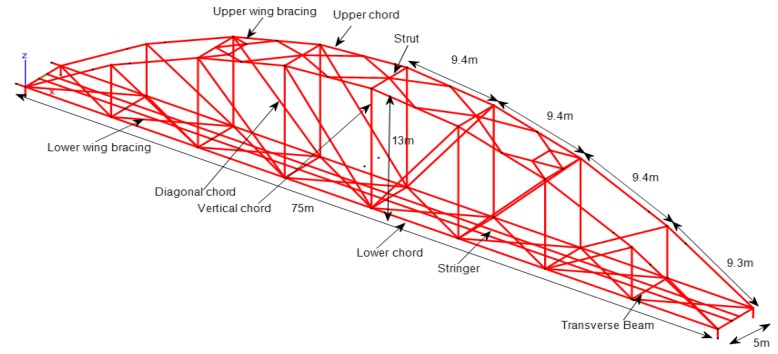
Main structural elements.

The truss members are made from steel with a variety of section types such as *I*, *L*, and Box (Figure 2), and connected to each other by bolts. 

The measured span (the first span from the Hai Van side) is put on rocker and pin bearings. Rocker bearings permit translation and rotation in one direction, while pin bearings only allow rotational movement. The characteristics of rocker and pin bearings (size, stiffness) are collected from catalogues of manufacturers to calculate the parameters of the equivalent springs. 

## 3. Experimental Measurements

### 3.1. The Ambient Vibration Test

#### 3.1.1. Test Description

**Figure 3:**
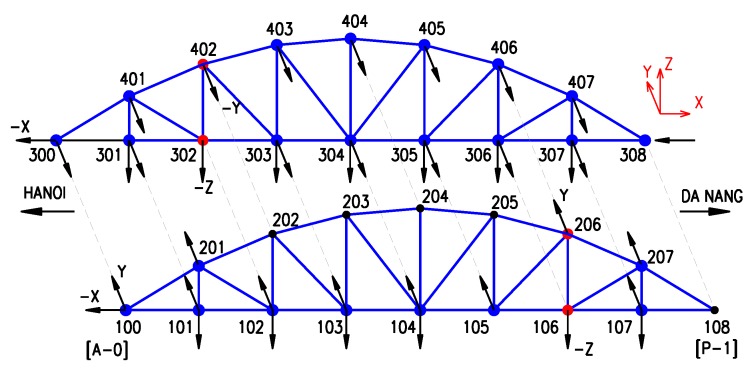
The measurement grid: accelerometers at 40 DOF; red: reference points–106, 206, 302, and 402; blue: roving points.

The modal identification test was performed on the first span between abutment *A-0* and pier *P-1*.The span length is *l* = 75 m; the maximum height at mid-span *h* = 13 m. In total, there are 32 truss connections. The dynamic response was due to ambient wind forces or the free vibration of the bridge after train passage. In order to obtain sufficient data for vibration-based system identification, as well as to be compatible with FE model analysis, ideally, all nodes and all directions (longitudinal-*x*, transversal-*y*, and vertical-*z*) from the bottom to the top of the arch-truss type should be included in the measurement grid. However, by neglecting deformation due to normal forces, several displacement components (degree of freedom or DOF) can be linked (slaved) to DOF of other (master) nodes. Therefore, in the measurement layout, 64 (=32 × 2) DOF are configured in either of two directions (*x* and *y*, or *y* and *z*, depending on the measurement positions: see Figure 3), of which 40 DOF are real measurements and 24 DOF are virtual (slave) results. An overview of the sensor layout is shown in Figure 3. 

#### 3.1.2. Sensor Placement

On the bridge, 10 accelerometers (PCB-393B12), with high sensitivity from 965 to 1083 mV/m/s^2^, were employed for response signal acquisition. However, the sensitivity of the accelerometers needs to be carefully considered in this case. It is well known that modal properties, especially the natural frequencies, are influenced by the environmental conditions, mainly the temperature. Therefore, the calibration is valid for the (constant) temperature during the ambient vibration test. If the measurements would be repeated as part of a SHM program, i.e., to detect structural damage, this dependency definitely has to be taken into account. In practice, this kind of bridge will have a high amplitude vibration during the train passage. Therefore, using a high sensitivity sensor can lead to distortion or clipping of the response. For this reason, besides the ambient response measurement, the vibration of the bridge is only considered after a train passage. The vibration measurement grid was divided into eight setups; each setup included a maximum of 10 accelerometers, as in Table 1. From these 10, four served as references, while the remaining accelerometers were roving over the bridge. The four reference sensors were present, placed at lower and upper nodes of the two bays (see Figure 3). An optimal reference is a sensor where all of the lower modes of vibration are present. Therefore, modeling the structure beforehand is a reliable basis for allocating where the reference sensors should be located. The division of sensors in “reference” and “roving” is necessary when the number of available sensors is lower than the number of DOF that need to be measured. In this case, a multi-setup measurement campaign is needed. Reference sensors are placed in nodes where all lower vibration modes have non-zero modal displacements. Here, the position was selected, based on the modal results of a preliminary FE model. It is also possible to generate the “optimal positions” of the reference sensors when applying the optimal sensor placement algorithms [29]. In practical measurements on bridges, there will be as many reference sensors as possible, dependent on the existing instrumentation. Preferably, there should be more than one reference sensor. In the case of multiple setups, the other roving sensors cover all of the remaining positions in the measurement grid.

In order to identify by model updating the real operational conditions of the bearings, five sensors were placed at bearings (four sensors at two nodes (100 and 300) in directions *x* and *y*, and the remaining one at node 308 in direction *x*). As the bearing at node 108 is a fixed one, no sensor was placed at this node.

#### 3.1.3. Data Acquisition Process

**Figure 4:**
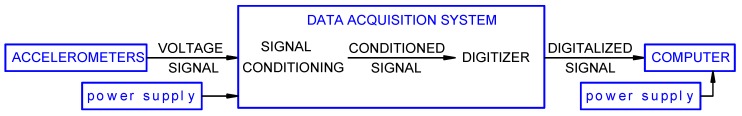
Data acquisition process.

A 12-channel data acquisition system, using three NI 9234 modules from National Instruments was employed to record the voltage signals from the sensors, and to convert these analog signals after conditioning to digital data (Figure 4). A portable computer is used to command the data acquisition system, and to read and save the digital data. 

**Figure 5:**
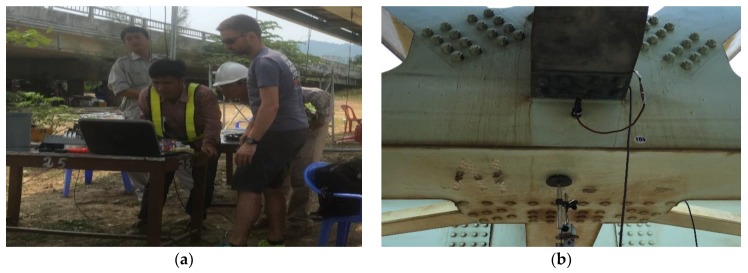
Field measurement instrumentation: (**a**) DAQ system (Compact DAQ Chassis NI 9178 and three vibration modules NI 9234) and portable computer; (**b**) Transversal accelerometer (PCB-393B12) at truss connection.

The total acquisition time was at least 10 to 20 min (approx. 900–1200 s) for one output-only setup at a sampling rate of 1651 Hz. It means that each channel has 1,485,900–1,981,200 data points. The envisaged acquisition time was 20 min for the ambient vibration test. For some setups, this was shortened because of the passage of a train during the measurement. However, a measurement duration of 10 min was considered to be sufficient, considering the frequencies of the lowest modes. As the system identification is done for each setup separately, the time lengths did not have to be identical. The measurement campaign took place in two successive days. Figure 5 shows the installation of the equipment on site.

### 3.2. System Identification by MACEC

#### 3.2.1. Data Pre-Processing

In this stage, MACEC software Edwin Reynders et al. [30] is employed to process the measured data. Some parameters in MACEC have to set up for treating the acquired acceleration data from eight setups in a systematic manner. The procedure of data pre-processing is as follows:

- The first step is to construct a grid of measured nodes, then connecting these nodes by lines to create a visualization of the structure.
- Input-required parameters e.g., labels, data types, sensitivities, amplification factors, and measurement units, corresponding to each channel.
- A pure ambient response measurement in a period of approximately 10 minutes is extracted.
- There is always an offset on the measured signal data; therefore, the DC component needs to be removed from the time series of all channels.
- A “FILT-FILT” function is applied to pass signals with a frequency higher than a cutoff frequency of 0.5 Hz, and to attenuate signals with frequencies lower than 0.5 Hz. The purpose of this step is to remove low-frequency blurring or noise by highlighting frequency trends, i.e., higher than 0.5 Hz [31].
- The frequency range of interest in the bridge structure often lies between 0–20 Hz. Therefore, to reduce the data and so on, to facilitate the System Identification, digital filtering is first applied to the measurement signals, followed by resampling them to achieve a Nyquist frequency of 20 Hz by the “DECIMATE” function with a decimation factor of 40.

#### 3.2.2. Covariance Based System Identification (SSI-COV)

After the pre-processing stage, a measurement model of structure will be identified. The stochastic subspace identification (SSI) method is often employed to perform system identification for the output-only or operational modal analysis (OMA) of structures. There exist two implementations of the SSI: the data-driven (SSI-data) option and the covariance (SSI-cov) option. [31] pointed out that the implementation of the SSI-cov is more straightforward, as well as being computationally less expensive than of the SSI-data. In comparison with SSI-data, the SSI-cov implementation also obtains a similar accuracy. Therefore, the dynamic system identification of the tested bridge was performed by SSI-cov.

The system identification was started, specifying the number of blocks that the raw time data was divided in. The number of blocks was used for computing the sample covariance of the output correlation matrices. In general, half the number of block rows *i*, can be chosen based on the relationship between the lowest frequency of interest and the Nyquist frequency. In practice, the value of *i* has a significant influence on the quality of the identified system model. Its value should be as large as possible; however, the excessiveness of the calculation time and memory usage should be considered [32]. For this case, the value of *i* is chosen as 250. 

Another parameter that needs to be considered is the maximum system order. In the theoretical aspect, observing the number of non-zero singular values of the block, Toeplitz can identify the system order *n*. In practice, it is not easy to inspect this number of non-zeroes; because of the noise from modelling inaccuracies, measurement noise…etc., the higher singular values do not exactly equal zero. Therefore, a maximal “gap” between two successive singular values becomes important for finding the system order. Peeters et al. [33] stated that the gap is not clear to find out, especially in large structures. For system identification of the Nam O bridge, the considered system order ranges from 2 to 140 in increasing steps of 2, i.e., [2:2:140]. 

### 3.3. Modal Analysis

**Figure 6:**
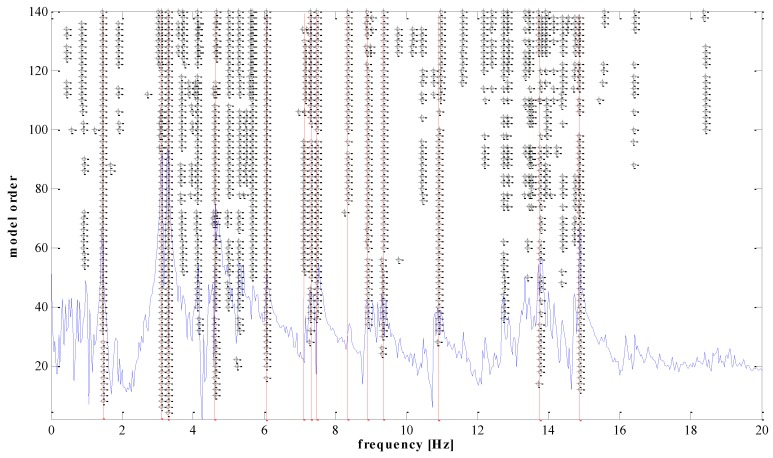
The stabilization diagram of setup 1 in the interval from 0 to 20 Hz.

To obtain a clear stabilization diagram when model orders range from 2 to 140, some criteria need to be specified. The criteria are: 1% for frequency stabilization, 5% for damping ratio stabilization, and 1% for mode shape stabilization. These values are selected based on experience with many other similar structures. The stable poles appear systematically in certain frequency sub-intervals, from 1 to 15 Hz. The stabilization diagram with the SSI-cov of setup 1 is shown in Figure 6 for illustration purposes.

**Figure 7:**
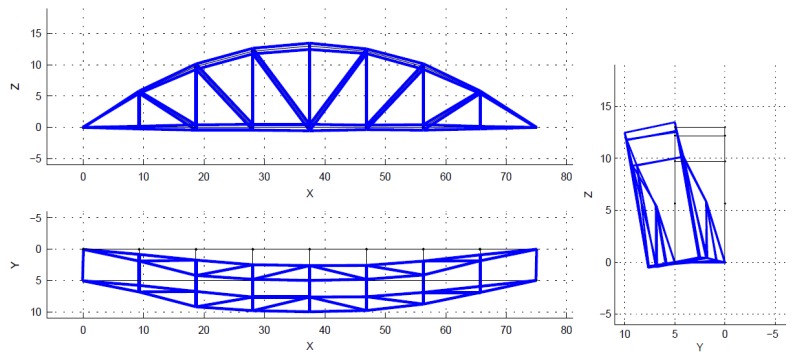
The identified mode 1, first lateral, f = 1.45 Hz, xi = 0.82%.

**Figure 8:**
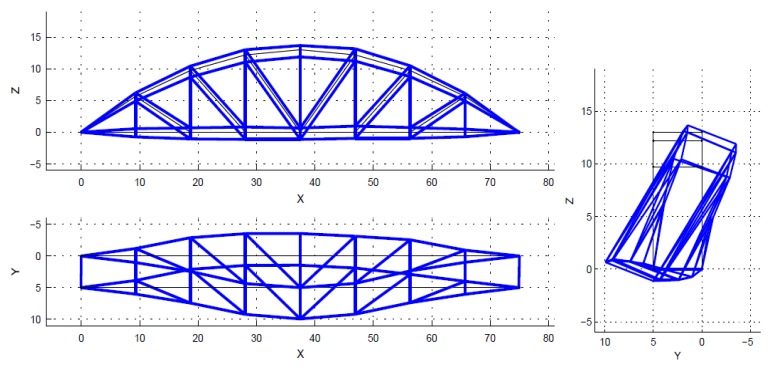
The identified mode 2, first torsion, f = 3.11 Hz, xi = 0.19%.

**Figure 9:**
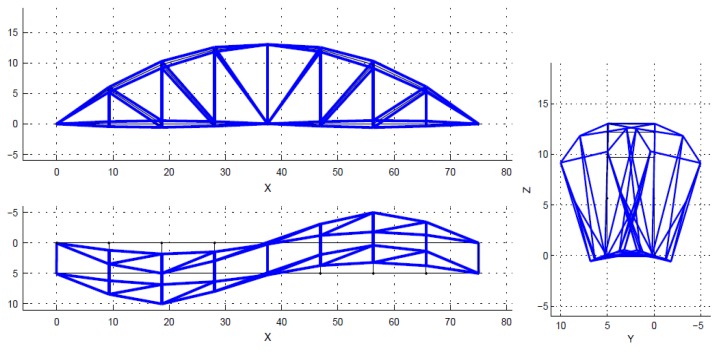
The identified mode 3, second lateral, f = 3.28 Hz, xi = 0.27%.

**Figure 10:**
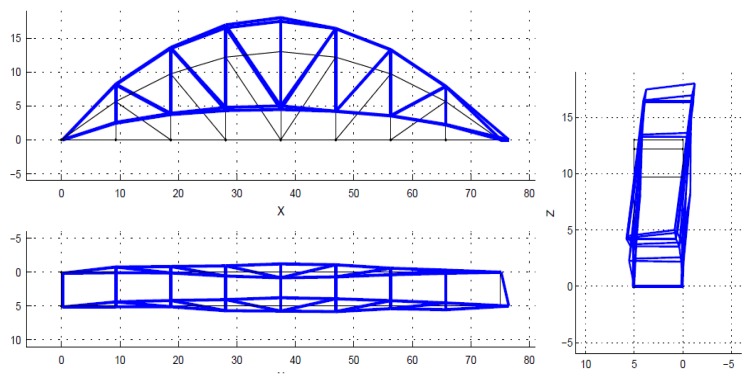
The identified mode 4, first vertical bending, f = 4.62 Hz, xi = 2.54%.

**Figure 11:**
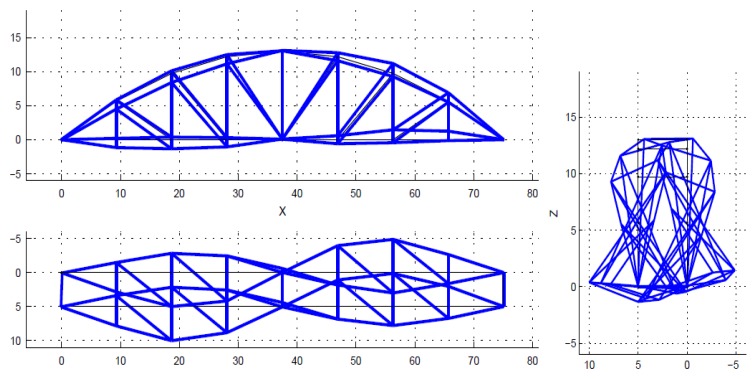
The identified mode 5, second torsion, f = 6.05 Hz, xi = 0.28%.

Theoretically, a bridge has a multitude of vibration modes. However, the modes of vibration do not contribute equally to the response of a structure. Natural frequencies and mode shapes are determined based on stable poles. The spectrum is just a visual aid, and is not used to select the poles. The poles (also closely spaced) are identified by the SSI algorithm in MACEC [30]. Normally only the first few modes, which have higher participation factors, are considered, in order to obtain the dynamic response of structures. Mainly, those main lower modes are enough to solve the model updating problem. In this case, only the first five modes were used within the frequency interval from 1.45 Hz to 6.05 Hz, as shown in Figure 7, Figure 8, Figure 9, Figure 10 and Figure 11. For a detailed explanation about the construction and the interpretation of the stabilization diagram in Figure 6, the reader is referred to [30,33].

## 4. Finite Element Model

**Figure 12:**
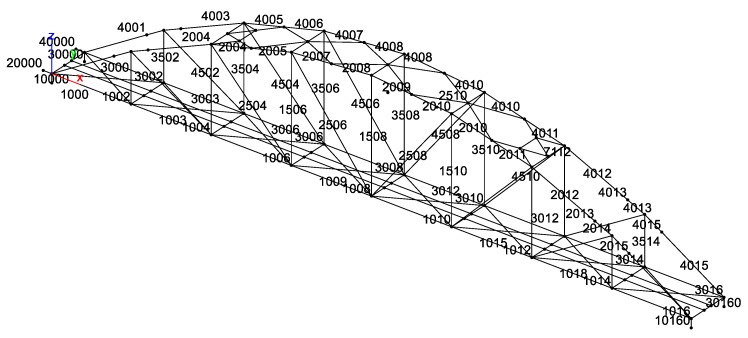
Finite element (FE) model of the Nam O Bridge.

In order to predict structural dynamic behavior, and compare it with that obtained from measurement, a finite element model of Nam O bridge was built by using the MATLAB toolbox StaBil [34] (see Figure 12).

The following details describe the FE model:

- The bridge is modeled by 137 nodes, 227 elements, and nine section types of truss members are used (see Table 2).
- Main structural members are shown in Figure 2: upper chords, lower chords, vertical chords, diagonal chords, stringers, upper wind bracings, lower wind bracings, and struts, are modeled using three dimensional (*3D*) beam elements. This element has six degrees of freedom at each node, including translations in the *x*, *y*, and *z* directions, and rotations around the *x*, *y*, and *z* directions. It is based on Timoshenko beam theory, which includes shear-deformation effects. The element provides options for unrestrained warping and restrained warping of cross-sections. The transverse girders (of the deck system) are also modeled using beam elements.
- The global *x*-axis is in the longitudinal direction of the bridge; the *z*-axis is in the transverse direction (to the river flow direction), and the *y*-axis is in the vertical direction. The left main truss is on the downstream side. Likewise, the right main truss is on the upstream side. Both trusses represent the side view of the bridge (Figure 1).
- Bearings were modeled by using spring elements.
- Mass is added to the lower chords: the rail track and non-structural components such as power lines, hand rails, maintenance paths, and protections were estimated at 247.2 km per meter.
- Section properties and material properties of structural members are listed in Table 2 and Table 3.

The connection between truss joints: due to the uncertainty of the actual joint stiffness, three possible models of link types are considered: (a) pin, (b) rigid, and (c) semi-rigid. This process can be considered as a trial step to analyze and determine some unknown parameters (uncertainties in material properties, section properties, or joint conditions, etc.). 

Case 1: Pin connection

**Figure 13:**
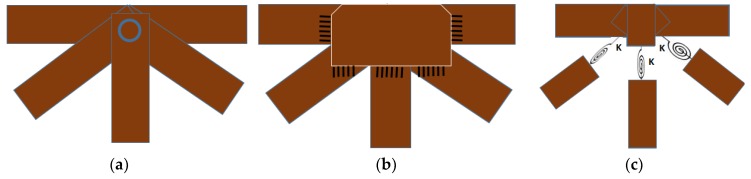
Connection types of truss joints: (**a**) Pin connection; (**b**) Rigid connection; (**c**) Semi-rigid connection (rotational springs).

In order to simplify the calculation, researchers as Duerr [35], Deng et al. [36], and Saka [37] assumed that truss members are linked to each other by pin connections (Figure 13a). In this case, the influence of the rotational stiffness is neglected, and no moments are transferred between the truss members. In a static analysis of truss structures, this link is often applied for the node joints, which have little effect on the result of the force of the truss members, because the moment transfer is insignificant with the axial forces (compression and tension). This is assuming that a pin connection results in a statically determinate structure that can easily be calculated by hand.

Case 2: Rigid connection.

Duerr [35], and other authors have also applied rigid links (Figure 13b) for truss joints. Basically, a rigid joint can transfer axial forces (compression, tension), as well as moments between members.

Case 3: Semi-rigid connection.

**Figure 14:**
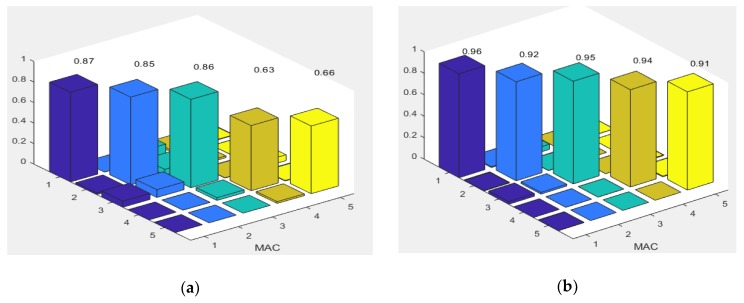
MAC values of mode shapes before model updating: (**a**) Rigid connection, (**b**) semi-rigid connection.

This link type has recently been applied by many researchers. Luong el al. [26] determined the stiffness of node joints, when updating a truss structure in laboratory, and found that a semi-rigid connection represent the most accurately the dynamic characteristics of the considered truss structure. Dubina et al. [27], Csébfalvi [28], and other researchers applied semi-rigid links (rotational springs) for truss structures. However, most of the aforementioned authors only applied this type of joint in static analysis, whereas a few others employed it to analyze the dynamic analysis of scale models in the laboratory. Rotational springs (Figure 13c) are often used to present semi-rigid links at truss joints. This study also applied the aforementioned three scenarios of joint conditions to predict structural dynamic behavior of the Nam O Bridge. Linear elastic rotational springs with three degrees of freedom at each node: rotations around the x, y, and z directions are used for vertical chords and diagonal chords, as in Figure 13c. The original stiffness of rotational springs is estimated according to reference [26]. The results are given in Table 4 and Figure 14.

Table 4 demonstrates that the FE model of a truss bridge with a pinned connection do not predict the behavior of the Bridge properly. The frequencies of the first five modes are lower than those of the measurement. The result of the FE model with rigid connection is also not satisfactory in comparison to the experimental one. Specifically, there are deviations between the frequencies calculated from FEM, and the measurements (from 26% to 36%). All frequencies from the FE model were higher than those from the experiment. Rigid links make the structure stiffer than in reality, while the truss members in Nam O Bridge are linked with each other by bolts. Additionally, MAC values (Figure 14a) that are lower than 0.9 indicate the difference between the mode shapes of the FE model, and the measurement. The model with a semi-rigid connection provides improved simulated modal results, in comparison to the experimental ones. There is a small deviation between the frequencies that are calculated from FEM, and the measurement (around 1%, apart from mode 5 having 13%). MAC values (Figure 14b) higher than 0.9 indicate a consistent correspondence between the numerical model and the measurement [38,39]. However, it is necessary to update some uncertain parameters, such as: Young’s modulus, and the stiffness of springs at bearings and truss joints to obtain the best correspondence between the theoretical and experimental results.

## 5. Model Updating

### 5.1. PSO Algorithm

In 1995, Eberhart et al. [40] developed particle swarm optimization (PSO) algorithm referenced from animal (particle) behaviors such as birds flocking and fish schooling. Each particle “flies” or “swarms” randomly through the search space, and records and communicates with other particles about the best solution (the best local position) that they have discovered. Therefore, it can assist in looking for the best global position (the best solution) easier and faster. PSO has already been applied in many optimization issues. Khatir et al. [41] used PSO to predict the location and severity of damage of a steel cantilever beam and a two-dimensional frame, based on the inverse problem. Wei et al. [42] employed an improved PSO to detect the damage of different structures (a frame, beam, and a truss) with a high accuracy. Shabbir et al. [43], Shao et al. [44], Qiing et al. [45], Pau et al. [46], Mangiatordi et al. [47] and other researchers also successfully used PSO to deal with other optimization problems. The PSO algorithm is based on two equations. The first equation updates the position of a particle:

*x^i^*(*t* + 1) = *x^i^*(*t*) + *v^i^*(*t* + 1), (1)

The second equation updates the velocity of a particle:

*v^i^*(*t* + 1) = *wv^i^* + *C*_1_*r*_1_(*p^i^*(*t*) − *x^i^*(*t*)) + *C*_2_*r*_2_(G_best_ − *x^i^*(*t*)), (2)

where *x^i^*(*t*), and *x^i^*(*t* + 1) represent the position vectors of particle *i* at time t and *t* + 1, respectively; *v* is the velocity vector of particle, *w* indicates the inertia weight parameter, *C*_1_, *C*_2_ represent the cognition learning factor and the social learning factor, respectively, *r*_1_ and *r*_2_ are random numbers in the range of (0,1), *p^i^*(*t*) is the best position of each particle (the local best), and G_best_ is the best position of all particles (the global best). Each particle is featured by a velocity vector and its physical position in the space. In the moving process, particles can remember the best local position *p^i^*(*t*), and communicate with others to look for the global best position (G_best_). The algorithm develops iterations to determine the fitness of each particle, based on the objective function. When the objective function is minimum, the global best position (G_best_) is achieved. Because only the best global position of particles (G_best_) is given out, with a few parameters to adjust, compared with other optimizations algorithms, PSO tends to converge to the global best faster, reduces the computational time, and results in a good correspondence between the FE model and the real structure.

### 5.2. Genetic Algorithm

The Genetic Algorithm (GA) is an evolutionary optimization method, used efficiently for different kind of optimization problems over the last few decades [48,49]. In our study, different individuals, also called chromosomes or populations, represent the rigidity of springs to update using the experimental results of natural frequencies and mode shapes. The population evolves iteratively towards a better solution, in a process inspired from a natural evolution, where they are allowed to cross among themselves in order to obtain favorable solutions. For a review of the approach that is searching for the global best GA, the reader is referred to [50]. The fitness is the objective function value, calculated in Equation (3). The best feasible solutions have higher probabilities of being chosen as parents of new individuals, where the properties of the parents are combined by exchanging chromosomes parts to produce two new designs. Afterwards, the mutation is performed by randomly replacing the digits of a randomly selected chromosome. These basic operators are repeated to create the next generations until the maximum number of iterations.

### 5.3. Model Updating of the Nam O Bridge

**Figure 15:**
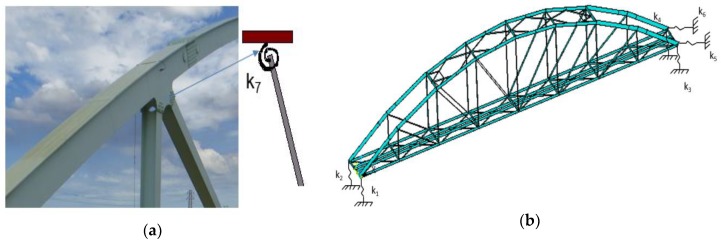
Uncertain structure parameters are selected to update in model: (**a**) The springs at truss joints; (**b**) The springs at bearings.

A FE model update was applied in the Nam O Bridge. Eight uncertain parameters, including Young’s modulus of truss members (E), and the stiffness of six springs under bearings (*k*_1_,*k*_2_,*k*_3_,*k*_4_,*k*_5_,*k*_6_) as shown in Figure 15, and the stiffness of rotational springs at truss joints (*k*_7_), were chosen for updating. The original stiffness of the rotational springs is estimated based on reference [26], whereas the stiffness of springs at the bearings is calculated based on the bearing types. The stiffness of springs is listed in Table 7.

The objective function was built, based on both the mode shapes and the natural frequencies as follows:

(3) $$\begin{array}{c} \mathrm{Fitness}=\sum_{k=1}^{5}\left[1-MAC\left(\varphi_{k},\tilde{\varphi_{k}}\right)\right]+\sum_{k=1}^{5}\frac{\Delta {\omega_{k}}^{2}}{{\tilde{\omega_{k}}}^{2}}=\sum_{k=1}^{5}\left[1-\frac{\left(\tilde{\varphi_{k}^{T}}\cdot \varphi_{k}\right)2}{(\varphi_{k}^{T}\cdot \varphi_{k})\cdot (\tilde{\varphi_{k}^{T}}\tilde{\cdot \varphi_{k})}}\right]\\ +\sum_{k=1}^{5}{\left(\omega_{k}-\tilde{\omega_{k}}\right)}^{2}/{\tilde{\omega_{k}}}^{2}, \end{array}$$

**Figure 16:**
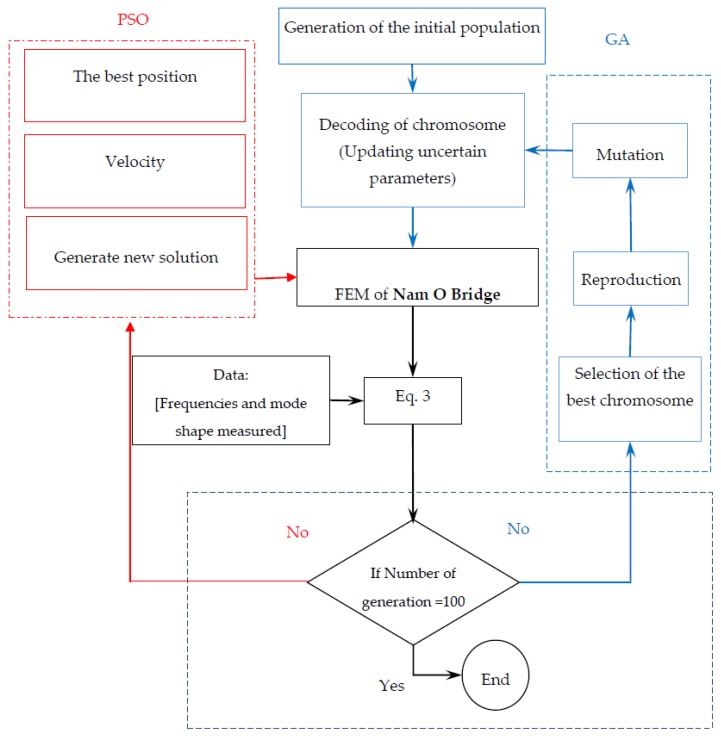
Methodological approach for model updating by using genetic algorithm (GA) and particle swarm optimization (PSO).

On the right hand side of Equation (3), the first part is the discrepancy between the first five mode shapes in terms of the MAC values, and the second part presents the deviation between the numerical and experimental first five natural frequencies. ($\varphi_{k},\omega_{k}),\;(\tilde{\varphi_{k}},\tilde{\omega_{k}}$) are analytical and experimental frequencies, mode shapes, respectively, “*k*” is the modal order, and *T* denotes a transposed matrix. The PSO and GA algorithm is utilized to look for the minimum (convergence) of the objective function (fitness), performed as illustrated by the diagram in Figure 16.

In PSO, a population size of 50 individuals is used. The inertia weight parameter (*w*) is 0.3, and the values of the cognition learning factor and the social learning factor are *C*_1_ = 2 and *C*_2_ = 2. In order to compare with PSO, for GA, the population size of 50 individuals is also applied; the crossover, and mutation coefficients are 0.8 and 0.1, respectively. The stop criteria of loops in both PSO and GA are established as follows: the deviation of objective function (fitness) value between two consecutive iterations is lower than 10^−7^, or the maximum number of iterations is 100.

**Figure 17:**
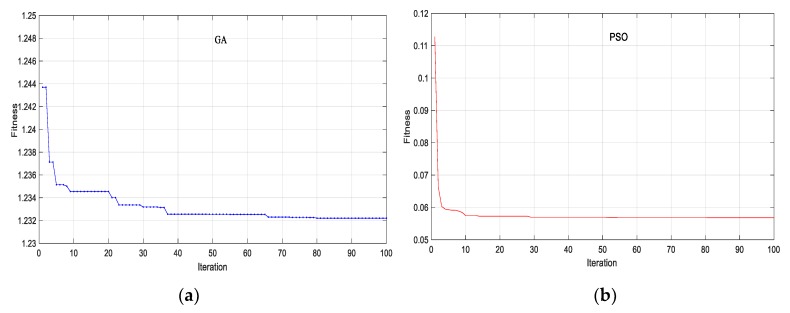
Fitness tolerance (**a**) GA; (**b**) PSO.

Figure 17 shows that the convergence rate of PSO is faster than GA. With the same population, PSO can find the result of the global best (the best solution) after only 10 iterations, Whereas GA needs approximately 38 iterations to obtain the global best. Besides, the convergence level of PSO outperforms than that of GA. The tolerance of objective function of PSO is lower 0.1, while the result of GA is about 1.2. That means that the deviation between numerical model and measurement after model updating using PSO was lower than GA. This result can be explained based on the approach to find the best solution in the two algorithms. While in PSO, only the best global position of particles (the best solution) is given out; in GA, information of all particles is shared with each other after an iteration. Therefore, the populations in PSO may not only look for the global best faster, but they may also avoid a local best.

### 5.4. Comparision between the Updated Model and Experimental Results

**Figure 18:**
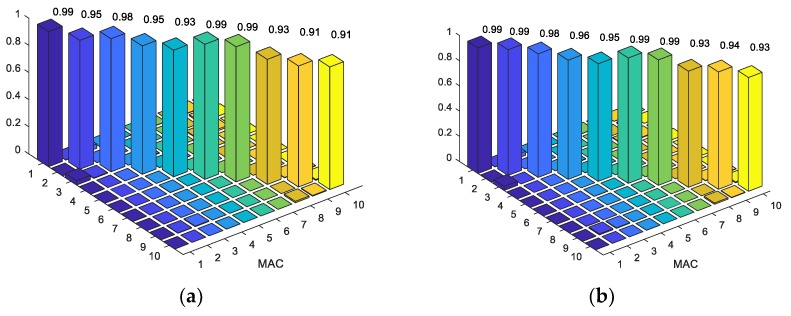
MAC values of mode shapes after model updating: (**a**) GA; (**b**) PSO.

A summary of the analysis and the experimental results is given in Table 5 and Figure 18. A good correspondence is obtained between the measured and the calculated frequencies and mode shapes of GA and PSO.

- The results of mode 1, mode 2, and mode 3 calculated by FEM and measurement to perfectly match.
- There are only small discrepancies in mode 4 and mode 5 (from 0.8% to 7.6%)
- The MAC values from 0.99 to 0.90 (Figure 18) demonstrate a close correspondence between the mode shapes of the FE model and measurement.
- The results of both calculated frequencies and mode shapes applying PSO are closer to those of the measurement than GA.
- After model updating, the FE model is applied to validate the higher modes that were not included in the objective function. Table 5 and Figure 18 show that the model updating also reduces the deviation between the measured and calculated frequencies, and the mode shapes of the higher modes (mode 6 to mode 10).

Table 6 provides the range of variation of the uncertainty parameters, based on experience, or estimated according to reference [26]. The model updating process also adjusts uncertain parameters of the bridge (Table 7). The changes in Table 7 show that the parameters before and after updating are not too much different. This is easy to explain, since the Nam O Bridge has been in operation for about seven years. Therefore, its properties still remain close to original. The stiffness of the bearing and rotational springs *k*_1_, *k*_2_, *k*_3_, *k*_4_, *k*_5_, *k*_6_, and *k*_7_ have a decreasing trend, with lower levels for GA compared to that of PSO. This proves that the stiffness of the bearings and rotational springs were overestimated. Therefore, to obtain a consistent correspondence between the theoretical and experimental results, the stiffness of the bearings and the rotational springs should decrease.

## 6. Conclusions

In this paper, an optimization-based FE model updating approach was applied to the Nam O Bridge, Vietnam. Trial FE (TEF) models (considering three scenarios of connection conditions of truss joints) are explored to obtain an accurate model, before applying GA and PSO algorithms for updating uncertain parameters. A close correspondence between the numerical and experimental results is achieved after model updating. The main conclusions can be summarized as follows:

- In order to obtain a high-fidelity FE model, the errors of the model structure must be reduced before the application of the model updating technique, instead of immediately changing the unknown parameters in the FE model. The TFE model was extremely vital in removing the relative deviation between the analytical and experimental results.
- For steel truss bridges, connections of truss members are the most complicated unknown parameters. The assumption of rigid or pinned joints has little effect on the result of static analysis. However, the result of dynamic analysis is extremely sensitive to this assumption. Semi-rigid (using rotational springs) joint conditions represent most accurately the dynamic characteristics of the considered truss bridge. Pin and rigid connections cannot exactly reflect the dynamic characteristics of the bridge, because while rigid links overestimate the stiffness of the joint, the pin links omit its stiffness.
- Mathematical power optimization algorithms such as GA and PSO, combined with an adequate FE model are powerful tools for solving optimization problems. PSO outperforms GA in terms of computational cost and accuracy.
- Further investigation should be considered, to employ evolutionary algorithms for model updating of more large-scale and complex structures, such as cable-stayed bridges or suspension bridges, all of which contain numerous uncertain parameters.

## Figures and Tables

**Table 1:** Overview of setups used for data acquisition and corresponding DOF.

Setup	Reference Channels				Roving Channels					
setup 1	106z	206y	302z	402y	101z	103z	301z	303z	305z	
setup 2	106z	206y	302z	402y	102z	104z	107z	304z	306z	307z
setup 3	106z	206y	302z	402y	102y	103y	104y	304y	306y	307y
setup 4	106z	206y	302z	402y	101y	105y	107y	301y	303y	305y
setup 5	106z	206y	302z	402y	102y	103y	104y	304y	306y	307y
setup 6	106z	206y	302z	402y	100x	100y	300y	300x	308x	
setup 7	106z	206y	302z	402y	403y	404y	405y	406y		
setup 8	106z	206y	302z	402y	201y	207y	401y	407y		

**Table 2:** Cross-sectional properties of truss members.

Member	Area(m^2^)	Moment of Inertia I_z_ (m^4^)	Moment of Inertia I_y_ (m^4^)
Upper chord	0.056	6.70 × 10^−04^	3.1 × 10^−03^
Lower chord	0.020	2.10 × 10^−04^	6.30 × 10^−04^
Vertical chord	0.010	5.49 × 10^−05^	1.15 × 10^−04^
Diagonal chord	0.014	1.24 × 10^−04^	2.78 × 10^−04^
Stringer	0.020	2.07 × 10^−04^	6.27 × 10^−04^
Transverse Beam	0.026	2.03 × 10^−04^	3.61 × 10^−03^
Strut	0.020	6.25 × 10^−04^	2.80 × 10^−03^
Upper wind bracing	0.0036	8.00 × 10^−06^	1.09 × 10^−05^
Lower wind bracing	0.0049	2.38 × 10^−06^	4.38 × 10^−06^

Note: I_y_ is the moment of inertia of the strong axis (the same direction with global Y), I_z_ is the moment of inertia of the weak axis (the same direction with global *Z*).

**Table 3:** Material properties of truss members.

Components	Value	Unit
Young’s modulus	200	GPa
Volumetric mass density	7850	Kg/m^3^
Poisson’s ratio	0.3	/

**Table 4:** The natural frequencies from the FE model for three connection cases, and from measurements.

Mode	Pin Connection (Hz)	Rigid Connection (Hz)	Semi-Rigid Connection (Hz)	Measurement (Hz)	Mode Type
1	1.18(18.6%)	2.05(29%)	1.47(1.4%)	1.45	First lateral
2	2.76(11.3%)	4.36(29%)	3.14(1%)	3.11	First torsion
3	3.11(5.18%)	4.44(26%)	3.32(1.2%)	3.28	Second lateral
4	3.79(17.7%)	7.18(36%)	4.80(3.7%)	4.62	First vertical bending
5	3.94(34.9%)	8.15(26%)	6.96(13%)	6.05	Second torsion

**Table 5:** The modal frequencies from the FE model after model updating compared to the measurement.

Mode	Before Model Updating (Hz)	Model Updating—GA (Hz)	Model Updating—PSO (Hz)	Measurement (Hz)
1	1.47(1.4%)	1.47(1.3%)	1.45(0%)	1.45
2	3.14(1%)	3.06(1.6%)	3.10(0.3%)	3.11
3	3.32(1.2%)	3.29(0.3%)	3.27(0.3%)	3.28
4	4.80(3.7%)	4.70(1.7%)	4.66(0.8%)	4.62
5	6.96(13%)	6.53(7.3%)	6.55(7.6%)	6.05
6	7.21(1.35%)	7.11(0.2%)	7.15 (0.4%)	7.12
7	7.50 (2.74%)	7.35(0.7%)	7.33 (0.5%)	7.30
8	8.33 (14,1)	8.21(10%)	8.10 (8.57%)	7.46
9	9.18 (10.81%)	9.05(9.2%)	9.00 (7.94%)	8.29
10	9.79 (10.16%)	9.64(8.5%)	9.57 (7.10%)	8.89

**Table 6:** The range of variation of the uncertainty parameters.

	*k*_1_	*k*_2_	*k*_3_	*k*_4_	*k*_5_	*k*_6_	*k*_7_	E
Lower	1.0	1.0	1.0	1.0	1.0	1.0	7	1.9
Upper	2.0	2.0	2.0	2.0	2.0	2.0	9	2.2

Note: the unit of *k*_1_,*k*_2_,*k*_3_,*k*_4_, is 10^10^ N/m, unit of *k*_5_,*k*_6_ is 10^7^ N/m, the unit of *k*_7_ is 10^5^ N.m/rad, and the unit of E is GPa.

**Table 7:** Value of uncertain parameters before and after updating.

	*k*_1_	*k*_2_	*k*_3_	*k*_4_	*k*_5_	*k*_6_	*k*_7_	E
Before	1.3	1.3	1.3	1.3	1.5	1.5	8	2
After(GA)	1.27	1.22	1.19	1.21	1.45	1.38	7.8	1.99
After(PSO)	1.20	1.16	1.12	1.16	1.40	1.33	7.6	1.98

Note: the unit of *k*_1_,*k*_2_,*k*_3_,*k*_4_, is 10^10^ N/m, the unit of *k*_5_,*k*_6_ is 10^7^ N/m, the unit of *k*_7_ is 10^5^ N·m/rad, and the unit of E is GPa.
